# In Vitro Cytotoxicity and Inflammatory Response of Local Anaesthetics and Corticosteroids in Canine Fibroblast-Like Synoviocytes

**DOI:** 10.1177/19476035261459650

**Published:** 2026-06-22

**Authors:** Catarina Sousa, Ana Lima, Adriana Belas, Sónia Campos

**Affiliations:** 1 I-MVET (Research in Veterinary Medicine), Faculty of Veterinary Medicine, 70887 Lusófona University – Lisbon University Center, Lisbon, Portugal; 2 School of Health, Protection and Animal Welfare, IPLUSO – Polytechnic Institute of Lusofonia, Lisbon, Portugal; 3 CECAV – Center of Animal and Veterinary Science, Faculty of Veterinary Medicine, Lusófona University – Lisbon University Center, Lisbon, Portugal

**Keywords:** osteoarthritis, fibroblast-like synoviocytes, local anaesthetics, cytotoxicity, corticosteroids, IL-6, MMP-9

## Abstract

**Objective:**

This study investigated the dose-dependent cytotoxicity and immunomodulatory effects of intra-articular local anaesthetics (lidocaine, mepivacaine, levobupivacaine) and corticosteroids (dexamethasone, methylprednisolone, methylprednisolone acetate) on canine fibroblast-like synoviocytes (FLS) *in vitro*. We evaluated cell viability and the expression of IL-6 and MMP-9.

**Methods:**

Canine FLS were exposed to increasing concentrations of intra-articular agents. Cell viability was determined via MTT assays. Immunomodulatory effects were assessed via ELISA for IL-6 and gelatin zymography for MMP-9 activity after TNF-α stimulation with IC_50_ concentrations.

**Results:**

All agents exhibited dose-dependent cytotoxicity. Dexamethasone was most cytotoxic (IC_50_ ≈ 3.4×10^-4^ mg/mL) but selectively inhibited TNF-α-induced MMP-9 activity without affecting IL-6 or inducing cytotoxicity at its IC_50_. Mepivacaine significantly inhibited MMP-9 activity by 40% at 1 mg/mL and uniquely induced IL-6 in unstimulated cells. Lidocaine, methylprednisolone, and methylprednisolone acetate suppressed TNF-α-induced IL-6 expression.

**Conclusions:**

Commonly used intra-articular agents induce dose-dependent cytotoxic effects on canine FLS at clinically relevant concentrations. This study provides the first evidence of species-specific responses for mepivacaine, levobupivacaine, and dexamethasone in canine FLS, highlighting mediator-specific and concentration-dependent effects crucial for optimizing intra-articular therapies in veterinary and translational medicine.

## Introduction

Degenerative processes of the cartilage are common in both humans and veterinary species, mainly represented by osteoarthritis (OA), a globally prevalent disease that is estimated to occur in 7.7%^1^ of the total human population and up to 65% of dogs during their lifetime.^2^ OA is a chronic and progressive disease characterized by cartilage degeneration, bone remodelling, and fibrosis of soft tissues, resulting in pain, inflammation, and reduced mobility.^3,4^ Although available treatments can alleviate symptoms, disease progression often leads to severe complications,^3^further research into effective OA treatments is essential for advancing medical therapies, with translational models benefiting both human and veterinary medicine.^5,6^ Given the strong similarities in pathophysiology, clinical manifestations and disease progression between humans and dogs, canine OA represents a valuable translational model for studying the mechanisms and potential treatments of this disease.^6-8^

Fibroblast-like synoviocytes (FLS) have been recognized as key factors in the pathogenesis of OA by promoting the production of pro-inflammatory cytokines such as TNF-α and IL-6 which can up-regulate matrix metalloproteinases, particularly MMP-9 (Gelatinase-B)^9,10^ exacerbating synovial inflammation and initiating a vicious cycle leading to cartilage degradation and joint stiffness.^4^ Consequently, targeting FLS toxicity, inflammatory markers and MMP-9 activity has become essential to identify therapeutic targets in order to reduce OA progression and improve joint health.^11^

Local anaesthetics (LAs) and corticosteroids are often administrated as intra-articular injections for both diagnostic and treatment of inflammatory and arthritic joint conditions in human and veterinary clinical practice.^12-14^ Some of the applications for intra-articular LAs and corticosteroids in veterinary species include diagnosis of lameness and joint diseases, anaesthesia during surgery, and joint pain management.^15,16^ Despite the widespread application of these drugs, there has been a growing concern for the potential toxicity over articular joint cells.^12^Recent studies have demonstrated the cytotoxic effect in chondrocytes and synovial fibroblasts in both *in vitro* human cells and animal models.^13,14^Both these drug classes can cause significant, dose and time-dependent cell death and metabolic dysfunction in joint tissues, especially at higher concentrations or with repeated exposure^.15,16^Nevertheless, LAs and corticosteroids are essential in clinical pain management, making it vital to understand their clinical and biomolecular effects on cell viability in order to explore strategies to prevent or reduce their cytotoxicity.^17^ Beyond their anaesthetic properties, LAs also possess significant anti-inflammatory effects by decreasing the expression of pro-inflammatory cytokines such as TNF- α and IL-6 and promote anti-inflammatory cytokines such as IL-10.^18-20^ Lidocaine has exhibited MMP-9 inhibition in inflammatory models by suppressing TLR4/NF-κB signalling pathway,^21,22^ although the direct effects on MMP-9 activity and expression remain poorly characterized, particularly in the context of OA. Studies with mepivacaine and levobupivacaine suggest that these agents modulate the inflammatory milieu promoting anti-inflammatory cytokines, potentially impacting MMP-9/TIMP-1 balance.^23,24^However direct evidence of MMP-9 inhibition is lacking, and these effects have not been specifically evaluated in OA-derived FLS. Similarly, corticosteroids are potent anti-inflammatory agents, dexamethasone can effectively inhibit catabolic marker gene expressions including MMP-9 and MMP-13 while significantly reduce IL-6 and IL-8 expression in human^25,26^ and rabbit FLS.^27^ Previous studies with methylprednisolone in human meniscal and synovial cultures revealed a significant suppression of MMP-2 and MMP-9^28^ and in equine cartilage explants both dexamethasone and methylprednisolone reduced interleukins expression and downregulated MMPs.^29^ However, current evidence of corticosteroids effects of MMP-9 in OA lack direct inhibitory effects. Therefore, further investigation is warranted to comprehensively assess both cytotoxic and biomolecular effects of these agents in clinically relevant translational models. Studies regarding the cytotoxic effects of these intra-articular agents remain limited, particularly towards canine FLS as, to the best of the authors knowledge, there is no direct studies specially of mepivacaine, levobupivacaine, dexamethasone and methylprednisolone. Based on these studies it is important to understand the direct cytotoxicity of these intra-articular drugs on articular cells, particularly FLS due to their role in OA inflammation,^11^ while also explore the impact of their combinations, varying concentrations and exposure duration on the joint’s inflammatory mileu.^4^ In this context, this study aims to evaluate the toxicity effect of different concentrations of lidocaine, mepivacaine, levobupivacaine, dexamethasone, methylprednisolone and methylprednisolone acetate on healthy canine FLS *in vitro*, and assess their impact on key inflammatory mediators IL-6 and MMP-9 after *in vitro* inflammation induction with TNF- α.

## Methods

### Isolation and Culture of FLSs From Synovial Fluid

Synoviocytes were originated from three adult canine patients. The animals were anaesthetised for musculoskeletal imaging diagnostics and for spaying surgery. Physical examination and pre-anaesthetic blood work (hemogram, leukogram and biochemistry) were within the normal values for the species. Animals were classified as ASA I. Under general anaesthesia, both metacarpal joint areas were clipped and followed by aseptic technique. Using a 22G sterile needle, both joints were punctured and an approximately 0.3 – 0.5 mL of synovial fluid was obtained from each joint samples from the joints, taken using syringes under aseptic conditions. For clinical purposes, samples were also collected for microbiology testing which came back negative for all animals. Procedures were conducted according to the best standards of care and guidelines for anaesthesia and surgery of dogs and after the owner and the ethics committee consents. Only proven normal FLSs samples was used in this study. To isolate FLSs from the fresh synovial fluids, each synovial fluid sample were resuspended in Dulbecco’s Modified Eagle’s Medium high glucose (DMEM; Biowest, Nuaillé, France), supplemented with 10% fetal bovine serum (FBS; Gibco BRL, Grand Island, NY, USA), 1% penicillin streptomycin solution (Pen-strep; Sigma-Aldrich, St.Louis, MO,USA) and 1% L-glutamine (Glutamax; Gibco BRL, ,Grand Island, NY, USA) and were seeded into 24-well culture plates separately. The plates were placed in a humidified atmosphere with 5% carbon dioxide (CO_2_) at 37°C. Growth medium was changed every 2 to 3 days with fresh medium until FLSs reached confluence. Upon 80% confluence, cells were passaged using trypsin-EDTA solution (0.5% trypsin, 0.2% EDTA in PBS; Gibco BRL, Grand Island, NY, USA) and cultures from passage 3 were used for the subsequent experiments.

### RNA Extraction

Total RNA was extracted from fibroblast-like synoviocytes (FLS) derived from canine synovial fluid using the RNeasy Plus Micro Kit (Qiagen, Hilden, Germany) according to the manufacturer’s instructions. This kit includes a genomic DNA (gDNA) eliminator column to ensure the removal of contaminating DNA. RNA concentration and purity were assessed using a microvolume spectrophotometer (Spex NanoSNAP™, USA), with an A260/A280 ratio of approximately 2.0 considered indicative of high-quality RNA.

### Real Time Quantitative RT-qPCR Analysis

Quantitative real-time RT-qPCR was performed to characterize FLS cell lines using the iTaq Universal SYBR Green One-Step Kit (Bio-Rad, Hercules, CA, USA) on a Rotor-Gene Q (Qiagen, USA) PCR Detection SystemAmplification was performed to the manufacturer’s instructions, with an initial reverse transcription step at 50 °C for 10 minutes, followed by 40 cycles of 95 °C for 1 minute and 60 °C for 30 seconds. All reactions were performed in triplicate. Relative gene expression levels were determined using the 2^−ΔCt^ method, with glyceraldehyde-3-phosphate dehydrogenase (GAPDH) serving as the internal control for normalization for each cell line. The primers used in this study were selected from previously published studies and are specific for canine joint tissues (Table 1).

**Table 1.** Sequences of Primers for the Genes Evaluated in RT-qPCR

Category	Gene name	Symbol	Primer sequence (5′–3′)	Reference
Housekeeping	Glyceraldehyde-3-phosphate dehydrogenase	*GAPDH*	FW: AGTATGATTCTACCCACGGGGC	^30^
			RV: CGAAGTGGTGATGGGGATGACT	
FLS	Hyaluronan Synthase 1	*HAS1*	FW:CAGACACGCTGGTTCCAAAT	
			RV: GCATAGAAGAGCCGCCAACAC	
Fibroblasts	Vimentin	*VIM*	FW: GGAGCAGCAGAACAAGATCC	^31^
			RV: AGACGTGCCAAAGAAGCATT	
Tight and adherent junction proteins	Cadherin 1	*CDH1*	FW: GACCCAGTAACTAACGACG	^32^
			RV: CTTCATTCACATCTTCCACG	
	Occludin	*OCLN*	FW:CACTACTGTGTGGTGGATCC	
			RV: CCTTGTCCCACAATATATTCG	

### In Vitro FLS Drug Exposure Assay

FLS cells were seeded in 24 well-plates at an average cell density of 5.3 x10^5^ cells per well for 24hr and then exposed to different concentrations of lidocaine, mepivacaine, levobupivacaine, dexamethasone, methylprednisolone and methylprednisolone acetate (Table 2) for another 24hr. The concentration range applied for each treatment was selected on the basis of commonly administrated intra-articular doses and previously published *in vitro* studies with animal and human cartilage cells. The cell media was then collected and stored at -80°C for further analysis. The experiment was performed in triplicates.

**Table 2.** List of *in Vitro* Treatments

Treatment	Concentration (mg/mL)	Reference dose (mg/kg)	Dose comparison (mg/kg)
Lidocaine	0.5, 1, 5,10	2 – 4	0.05 – 1
Mepivacaine	0.5, 1, 2, 4	2 – 4	0.05 – 0.4
Levobupivacaine	0.05, 0.1, 0.3, 0.6	1 – 2	0.005 – 0.06
Dexamethasone	0.001, 0.01, 0.025, 0.05	0.1 – 0.2	0.0001 – 0.005
Methylprednisolone	0.5,1,5,10	1-2	0.05 - 1
Methylprednisolone acetate	0.5,1,5,10	1-2	0.05 - 1

### TNF- α Inflammation Induction in FLS

FLS cells were seeded in 24 well-plates at an average cell density of 5.3 x10^5^ cells per well and allowed to adhere. Cells were then stimulated with 10 ng/mL TNF-α for 24hr to induce inflammatory response and then exposed for 24hr to the tested intra-articular drugs at concentrations based on IC_50_ values determined in the MTT assay, namely 0.1 mg/mL levobupivacaine, 1mg/mL mepivacaine, 3mg/mL lidocaine, 3.0 ×10^-4^ mg/mL dexamethasone, 1.0 mg/mL methylprednisolone and 0.5 mg/mL methylprednisolone acetate. Control groups without TNF-α stimulation were included and treated with the same compound concentrations to assess the effects of the compounds in non-inflammatory conditions. Cell media was then collected and stored at -80°C for further analysis. The experiment was performed in triplicates.

### MTT Assay

The 3-(4,5-dimehylthiazolyl-2)-2,5-diphenyltetrazolium bromide (MTT) assay was used to evaluate the effects of LAs and corticosteroids on mitochondrial dehydrogenase activity. FLS were seeded in 24-well plates (Corning) at an average density of 5.3 x10^5^ per well for 24hr and treated as described above for both the drug exposure assay and TNF-α stimulation. After 24hr, the culture medium was removed and the cells were washed twice with PBS, then 100μl of MTT (MTT; Sigma-Aldrich, St. Louis, MO, USA) working solution (5 mg/mL) was added into each well for a final concentration of 0.5 mg/mL per well. The culture plates were then incubated for 4 h at 37°C and 5% CO_2_. The MTT solution was then removed from the wells and MTT formazan crystals were dissolved with 100 μL dimethyl sulfoxide (DMSO; Sigma-Aldrich, St. Louis, Missouri, USA). The absorbance from each well was measured using a microplate reader (Thermo Scientific, Vantaa, Finland) at 570 nm. The experiment was performed in triplicates.

### Gelatin Zymography

To determine MMP-9 activity in the cell culture supernatants, a gelatin-zymography was performed using Novex 10% Zymogram Plus (Gelatin) protein gels (1.0 mm, 10-well; Thermo Fisher Scientific, Waltham, MA, USA), according to the manufacturer’s instructions. The cell culture supernatants were treated with nonreducing buffer (Laemmli Sample Buffer; Bio-Rad, Hercules, CA, USA) and 5 μl loaded into each well. After electrophoresis, gels were washed three times in 2.5% (v/v) Triton X-100 (Sigma-Aldrich, St. Louis, MO, USA), for 30 min each to remove the SDS and incubated 48 hr with zymogram developing buffer (Zymogram Developing Buffer; Novex, Thermo Fisher Scientific, Waltham, MA, USA). After incubation, zymograms were stained with Coomassie Brilliant Blue G-250 (Xpert Safe Protein Stain; GRISP, Porto, Portugal). White bands, visible against a blue background, marked the gelatinase activity of each proteinase. The experiment was performed in triplicates and protein band intensities were determined by densitometry analysis.

### Enzyme-Linked Immuno Sorbent Assay (ELISA)

The concentration of IL-6 in the cell culture supernatants was determined using a commercial ELISA kit (Canine IL-6 ELISA kit, FineTest, Wuhan, China), according to the manufacturer’s instructions. The experiment was performed in triplicates and IL-6 concentrations were normalized to the respective control group and presented as percentage values relative to control.

### Statistical Analysis

All experiments were performed in triplicates, at least three independent times and the data were expressed as the mean standard deviation (±SD). GraphPad Prism software (version 10.6.1) was used for comparing different treatments, using one-way analysis of variance (ANOVA). Dunnet’s test was used to compare differences between groups and the statistical differences with P value less than 0.001 were considered statistically significant. Dose–response curves were fitted to a three-parameter logistic model using GraphPad Prism and IC_50_ values were estimated from nonlinear regression, with 95% confidence intervals derived from the curve fit.

## Results

### FLS Lines Characterization and Validation

Quantitative real time PCR (qPCR) was performed to characterize and validate the three FLS cell lines derived from synovial fluid of three different canine patients (FLS A, FLS B and FLS C). The expression levels of key marker genes vimentin (VIM), E-cadherin (CDH1), occludin (OCLN), hyaluronan synthase 1 (HAS) were analysed relative to the housekeeping gene GAPDH. The cycle threshold (Ct) values, variance of gene expression between target gene and housekeeping gene GAPDH (ΔCt) and relative expression (2^−ΔCt^) are summarized in Table 3.

**Table 3.** Gene Expression Levels of the FLS Cell Lines (A, B and C) Relative to GAPDH

Gene	*FLS A*			*FLS B*			*FLS C*		
	*Ct*	ΔCt	2^−ΔCt^	*Ct*	ΔCt	2^−ΔCt^	*Ct*	ΔCt	2^−ΔCt^
GAPDH	13.99±0.20	-	-	13.26±0.16	-	-	18.26±0.18	-	-
VIM	17.30±0.97	3.31±0.10	0.10±0.04	16.99±0.16	3.74±0.17	0.07±0.03	25.82±0.45	7.56±0.25	0.01±0.01
CDH1	ND	-	-	ND	-	-	ND	-	-
OCLN	ND	-	-	ND	-	-	ND	-	-
HAS1	20.11±0.37	6.12±0.23	0.01±0.01	21.23±0.18	7.98±0.20	0.01±0.01	24.39±0.30	6.13±0.19	0.01±0.01

**ND**: not detected. Values represent means of at least 3 replicate experiments (n=3) ± SD.

All three FLS lines exhibited expression of VIM, a well-established intermediate filament protein characteristic of mesenchymal cells, including fibroblasts. The relatively low Ct values for VIM (ranging from 16.99 to 25.82) and detectable relative expression levels (0.01 to 0.10) across lines confirms their fibroblast-like nature. Similarly, the expression of HAS1 was consistent for the FLS A, B and C lines (0.01), with Ct values ranging from 20.11 to 24.39. Hyaluronan synthase 1 is a major component of extracellular matrix and synovial fluid, commonly expressed by synoviocytes. Its presence across cell lines indicates that they have retained a key functional characteristic of FLS. In contrast, epithelial market genes CDH1 and OCLN were not detected (ND) by qPCR indicating a lack of epithelial contamination in the cell cultures.

To assess the genetic similarity among the three canine FLS cell lines, one-way analysis of variance (ANOVA) was performed on the relative gene expression (2^−ΔCt^) values for the genes VIM and HAS1 with no significant differences across the three cell lines (p>0.05), indicating consistent gene expression of these two fibroblastic and synoviocytes markers.

### Effects of Local Anaesthetics and Corticosteroids on Canine FLS Cell Viability

**Figure 1.**
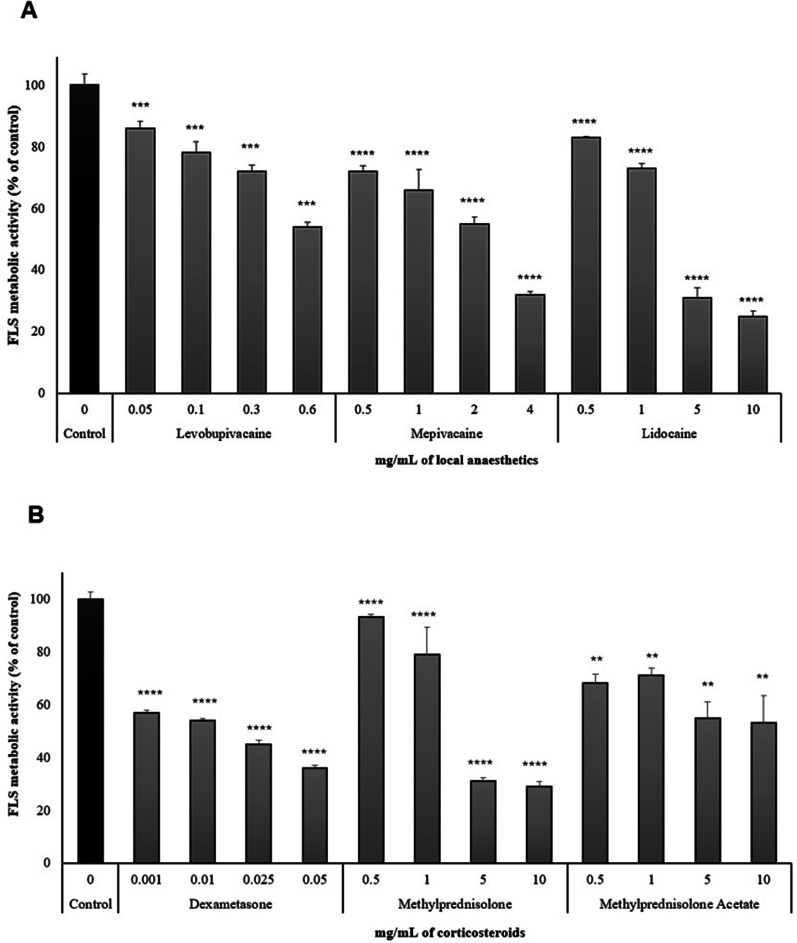
Cellular metabolic activity of canine FLS after 24hr exposure to the different treatments of local anaesthetics (A) and corticosteroids (B). Results are expressed as percentage of controls (no treatment). Graphic bars represent means of at least 3 replicate experiments (n=3) ± SD. Statistical analysis (one-way analysis of variance ANOVA) is represented by ** p<0.01, ***p<0.001 and ****p<0.0001 compared to control.

**Figure 2.**
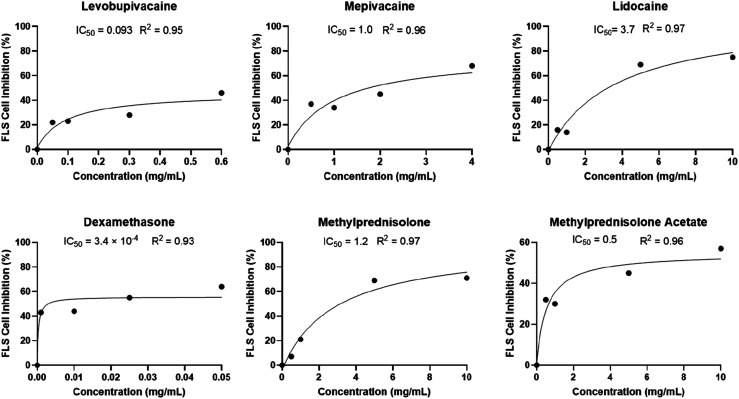
Dose–response curves showing the inhibitory effects of local anaesthetics and corticosteroids on canine FLS cell inhibition after 24hr exposure. Dose–response curves were fitted to a three-parameter logistic model using GraphPad Prism. IC_50_ values were estimated from nonlinear regression, with 95% confidence intervals derived from the curve fit. Data represents mean values from at least three independent experiments

The cytotoxic effects of lidocaine, mepivacaine, levobupivacaine, dexamethasone, methylprednisolone and methylprednisolone acetate on canine fibroblast-like synoviocytes (FLS) were evaluated after 24hr of exposure using MTT cellular metabolic activity assay. All compounds induced a concentration-dependent decrease in cellular metabolic activity (Figures 1A and B) consistent with a sigmoidal dose–response relationship (Figure 2).

Results from Figure 1A and B indicate a significant difference among all treatment groups compared to the control (p<0.001; p<0.0001), particularly dexamethasone with a significant dose-dependent cell viability reduction compared to control (p<0.0001) with the lowest concentrations. Methylprednisolone showed a marked decrease in metabolic activity (p<0.0001 for 0.5, 1, 5, and 10 mg/mL compared to control). In contrast, methylprednisolone acetate exhibited less cytotoxicity with less significant differences compared to control (p<0.01). The local anaesthetics had a significant dose-dependent metabolism decrease compared to the control (levobupivacaine p<0.001; mepivacaine and lidocaine p<0.0001) where FLS metabolic activity remained relatively higher in the presence of levobupivacaine.

Results from Figure 2 demonstrate that lidocaine, mepivacaine, and levobupivacaine exhibited concentration-dependent cytotoxicity toward canine FLS cells, with IC_50_ values ranging from 0.093 mg/mL to 3.7 mg/mL. Among the tested local anaesthetics, levobupivacaine exhibited the lowest IC_50_ of approximately 0.093 mg/mL, followed by mepivacaine (IC_50_ ≈ 1.0 mg/mL) and lidocaine (IC_50_ ≈ 3.7 mg/mL). Methylprednisolone exhibited an IC_50_ ≈ 1.2 mg/mL while methylprednisolone acetate exhibited a lower IC_50_ ≈ 0.5 mg/mL. Dexamethasone showed the highest cytotoxicity within the tested range, with IC_50_ ≈ 3.4×10^-4^ mg/mL but a plateau below 60 % inhibition.

**Figure 3.**
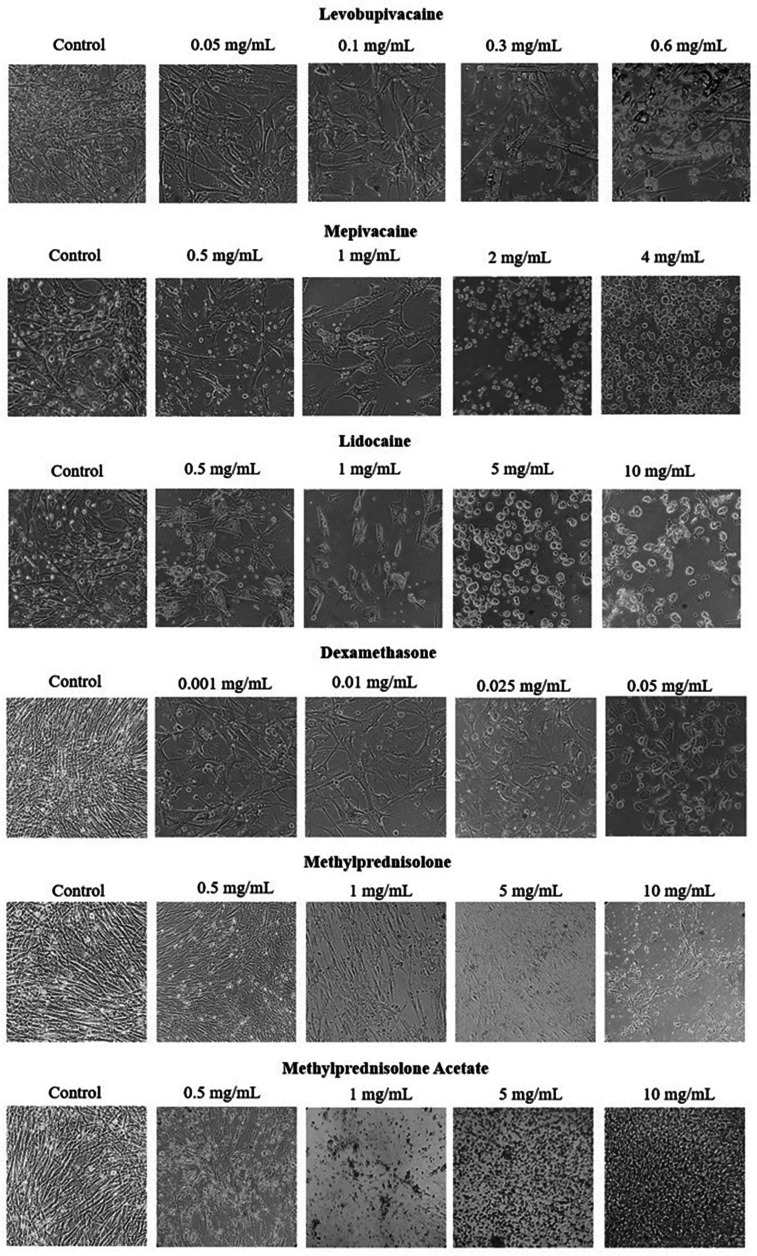
Representative light microscopy images illustrating morphological alterations of canine FLS after 24hr exposure to increasing concentrations of LAs and corticosteroids

Control cells displayed a confluent monolayer with typical spindle-shaped morphology, represented in Figure 3. Exposure to local anaesthetics and corticosteroids induced a dose-dependent reduction in confluence, accompanied by cellular rounding, detachment, and loss of integrity at higher concentrations. Among the agents tested, lidocaine and mepivacaine exhibited marked cytotoxic effects at ≥1 mg/mL, while levobupivacaine showed milder morphological changes, which became evident only at the highest concentrations tested. Dexamethasone exhibited a clear loss of cellular confluence starting from the lowest concentrations and a more accentuated morphological changes at ≥ 0.025 mg/mL. Methylprednisolone showed mild morphological changes at 5mg/mL with more pronounced effects at 10mg/mL, characterized by reduced confluence and increased cellular rounding and detachment. However, methylprednisolone acetate displayed significant cytotoxicity starting from 1mg/mL represented by loss of cell integrity and rounding and a significant decrease of confluence, becoming very pronounced at the highest concentrations.

**Figure 4.**
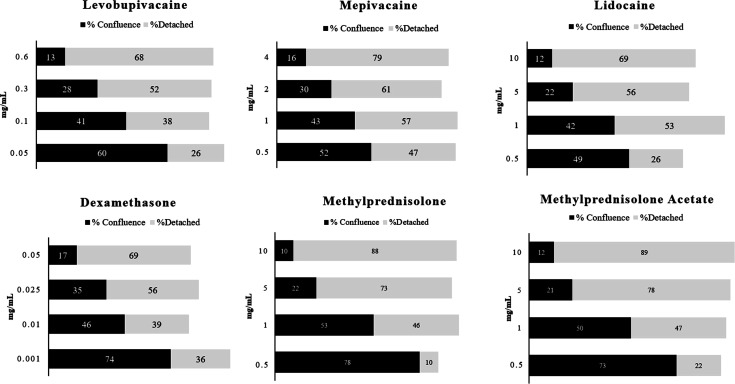
Quantitative analysis of FLS confluence and detachment after 24hr exposure to increasing concentrations of lidocaine, mepivacaine, levobupivacaine, and dexamethasone. Values represent the percentage of area covered by cells (% confluence) and the percentage of detached (non-viable) particles quantified by ImageJ particle analysis

The quantitative analysis of FLS viability (Table 4) and confluence (Figure 4) is consistent with cytotoxicity effects of each agent obtained in Figure 1, with a concentration-dependent decrease in the percentage of viable (attached) cells, accompanied by a corresponding increase in non-viable (detached) cells, was observed across all treatments, alongside with the respective loss of confluence.

**Table 4.** Quantification of Viable (Attached) and Non-viable (Detached) FLS Cells Following 24hr Exposure to Each Treatment. Cell Counts Were Obtained Using Fiji (ImageJ) as Number of Particles Analysed. Attached (Viable) Cells Were Identified Using a Circularity Range of 0.0–0.7, While Detached (Non-viable) Cells Were Quantified With a Circularity Range of 0.8–1.0. Percentages Were Calculated Relative to Total Counted Cells $\%\;\text{Viable}=\frac{N\;\text{Viable}}{N\;\text{Viable}+N\;\text{Non}‐\text{viable}\;}\times 100$

Treatment	Concentration mg/mL	Viable cells ± SD	Non-viable cells ± SD	% Viable	% Non-viable
Control	0	1586 ± 6	85 ± 5	95	5
Levobupivacaine	0.05	1214 ± 16	415 ± 16	75	25
	0.1	908 ± 13	563 ± 15	62	38
	0.3	678 ± 12	730 ± 4	48	52
	0.6	431 ± 10	935 ± 10	32	68
Mepivacaine	0.5	676 ± 3	601 ± 11	53	47
	1	610 ± 3	803 ± 6	43	57
	2	575 ±4	893 ± 9	39	61
	4	323 ± 12	1218 ± 12	21	79
Lidocaine	0.5	1104 ± 2	386 ± 8	74	26
	1	543 ± 5	603 ± 10	47	53
	5	507 ± 9	657 ± 5	44	56
	10	366 ± 5	804 ± 11	31	69
Dexamethasone	0.001	1014 ± 12	562 ± 10	64	36
	0.01	986 ± 9	636 ± 7	61	39
	0.025	567 ± 9	719 ± 15	44	56
	0.05	354 ± 7	779 ± 16	31	69
Methylprednisolone	0.5	1436±5	89 ± 3	94	6
	1	822 ±8	269 ± 6	75	25
	5	507± 10	495 ± 7	51	49
	10	414 ± 9	519 ± 11	44	56
Methylprednisolone Acetate	0.5	1427±3	195±8	88	12
	1	624±7	299 ± 14	68	32
	5	363 ±9	636 ±7	36	64
	10	297 ± 13	958±5	24	76

### FLS Inflammatory Induction by TNF-α

**Figure 5.**
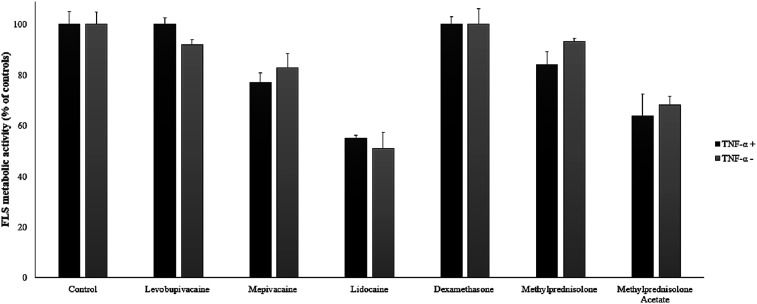
Cellular metabolic activity of canine FLS after 24 hr exposure to the IC_50_ values of each LAs and corticosteroids, with previous TNF- α stimulation (TNF- α **+**) or no stimulation (TNF- α -). Results are expressed as percentage of controls (no treatment). Graphic bars represent means of at least 3 replicate experiments (n=3) ± SD. Statistical analysis (two-way analysis of variance ANOVA) was used to compare between TNF- α stimulation or absence (p>0.05)

**Figure 6.**
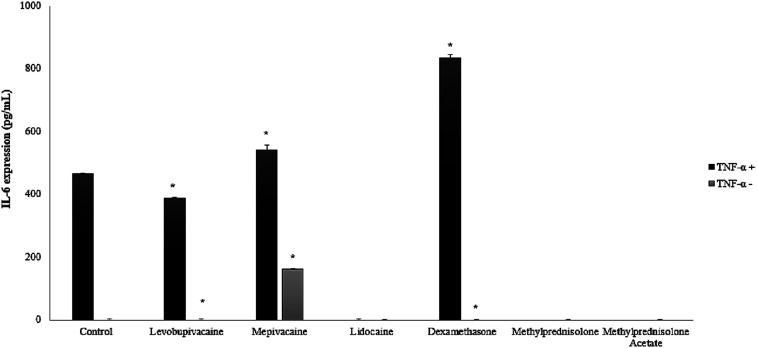
Effects of LAs and corticosteroids in IL-6 expression with TNF- α stimulation (TNF- α +) and without stimulation (TNF- α -), quantified by ELISA assay. Results are expressed as percentage of controls (no treatment). Graphic bars represent means of at least 3 replicate experiments (n=3) ± SD. Statistical analysis (two-way analysis of variance ANOVA) was used to compare between TNF- α stimulation or absence (*p<0.05)

**Figure 7.**
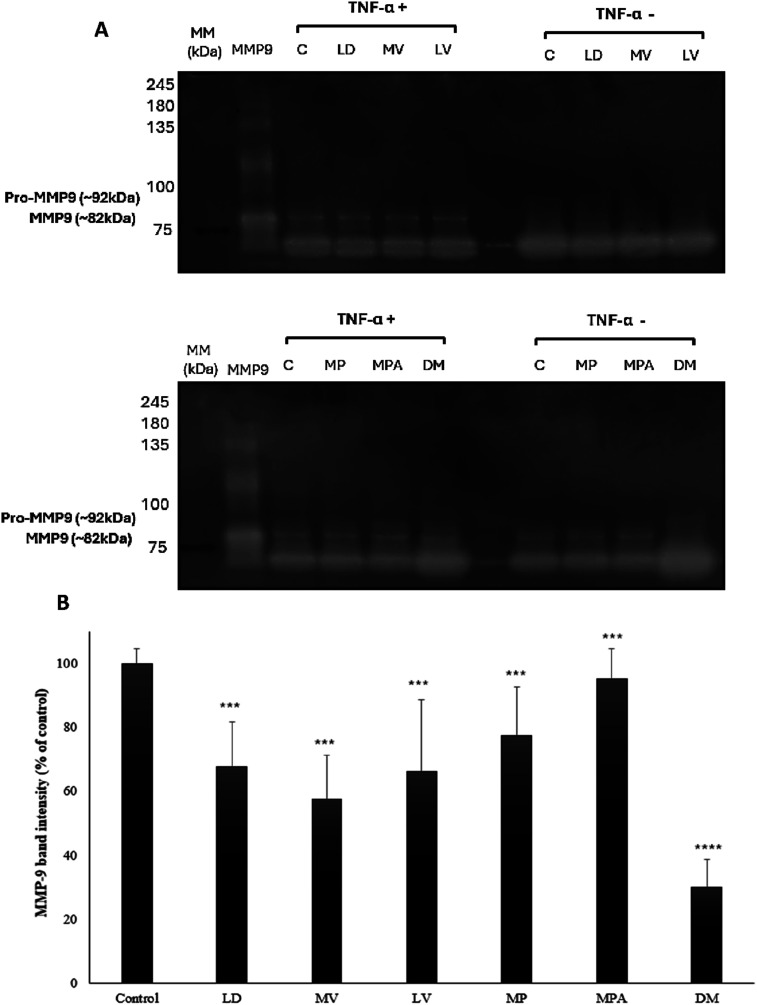
(A) Representative images of gelatin zymography analysis of MMP-9 activity in the cell culture supernatants of FLS exposure to lidocaine (LD), mepivacaine (MV), levobupivacaine (LV), methylprednisolone (MP), methylprednisolone acetate (MPA) and dexamethasone (DM), with TNF- α stimulation (TNF- α +) and without stimulation (TNF- α -). (B) Corresponding densitometry reading of bands through ImageJ software. Graphic bars represent means of at least 3 replicate experiments (n=3) ± SD. Statistical analysis (one-way analysis of variance ANOVA) is represented by ***p<0.001 and ****p<0.0001 compared to control

To evaluate the effect of inflammatory stimulation on FLS responses, cells were pre-exposed to 10 ng/mL TNF-α for 24hr to induce an inflammatory phenotype. Following this induction, cells were treated with each compound at their respective IC_50_ concentrations, namely 0.1 mg/mL levobupivacaine, 1mg/mL mepivacaine, 3mg/mL lidocaine, 3.0 ×10^-4^ mg/mL dexamethasone, 1mg/mL methylprednisolone and 0.5 mg/mL methylprednisolone acetate and their cytotoxicity was evaluated with and without TNF-α stimulation as represented in Figure 5. Expression levels of IL-6 were measured through ELISA (Figure 6) and MMP-9 activity was evaluated by gelatin zymography (Figure 7).

Results from Figure 5 indicate that all compounds were not cytotoxic at the concentrations tested, when comparing the metabolic activity of cells with and without TNF-α stimulation there was no significance (p>0.05). While generally safe, lidocaine and methylprednisolone acetate exhibited the lowest metabolic activity, approximately 60%, under these conditions, which was still considered within the non-cytotoxic range.

ELISA analysis of IL-6 expression revealed distinct patterns of modulation by the tested LAs and corticosteroids in the canine FLS cells, with significant differences between TNF- α stimulation and without stimulation, particular with levobupivacaine, mepivacaine and dexamethasone (p<0.05). In the absence of inflammation induction, mepivacaine led to an expression of IL-6 (162.51 pg/mL) in contrast with the rest of the tested compounds where no IL-6 was detected. Upon TNF- α stimulation, control cells exhibited a robust increase in IL-6 expression (465.36 pg/mL). Similarly, mepivacaine (542.56 pg/mL), levobupivacaine (389.57 pg/mL), and dexamethasone (835.23 pg/mL) also showed high levels of IL-6 expression in the presence of TNF-α. In contrast, lidocaine, methylprednisolone, and methylprednisolone acetate effectively suppressed IL-6 expression to undetectable levels in the presence of TNF-α stimulation.

Results from Figure 7 indicate that in the absence of TNF- α, minimal to no MMP-9 activity was detected, both pro-MMP-9 and active MMP-9, for all treatment groups including control. Due to these undetectable levels, densitometry analysis was exclusively performed on samples stimulated with TNF-α. (Figure 7B). Upon TNF- α stimulation, control cells exhibited prominent bands for both forms of MMP-9 which indicates that TNF-a stimulation positively induced MMP-9 activity in FLS cells (Figure 7A). Lidocaine (LD) significantly reduced MMP-9 activity to approximately 35% of the control (p<0.001) and similar effect was observed for levobupivacaine (LV; p<0.001) (Figure 7B). Mepivacaine (MV) demonstrated a stronger inhibitory effect by 50% compared to control (p<0.001). Methylprednisolone (MP) and methylprednisolone acetate (MPA) moderately reduced MMP-9 band intensity with approximately 20% and 10% inhibition respectively (p<0.001). Dexamethasone (DM) was the most potent inhibitor of MMP-9 activity by 70% compared to control (p<0.001).

## Discussion

In this study, three canine FLS lines were successfully isolated from synovial fluid samples of three different canine donors and demonstrated consistent fibroblast-like characteristics and synovial origin. Overall, our findings indicate that commonly used intra-articular agents exhibit dose-dependent cytotoxic effects on canine FLS, while also displaying distinct profiles in the modulation of inflammatory mediators. Expression of VIM and HAS1 across cell lines supports their functional relevance, while absence of epithelial markers CDH1 and OCLN excluded contamination, validating the experimental model.

Cytotoxicity assessment of the studied intra-articular agents revealed a clear dose-dependent cytotoxic response characterized by morphological alterations such as cell rounding and detachment. Our findings provide first direct evidence of mepivacaine, levobupivacaine, and dexamethasone-induced toxicity in canine synoviocytes, addressing a significant gap in the literature and highlighting potential species-specific responses. The selected concentration range aligns with commonly administrated intra-articular doses and established *in vitro* studies in cartilage cells, confirming the reproducibility and validity of our observed toxicity patterns.

Lidocaine, a widely used LA in OA treatment,^33^ exhibited cytotoxicity at 5 mg/mL (0.5%) reducing canine FLS cell viability by more than 50%, with an estimated IC_50_ of approximately 3.7 mg/mL, suggesting that up to 0.3% lidocaine would be considered safe. These findings align with previous *in vitro* studies in various joint cell types, where 1% lidocaine induced dose and time-dependent cytotoxicity in bovine articular chondrocytes,^34^ canine^33^ and human chondrocytes.^35^Studies with canine cartilage and synovial tissue cells also reported cytotoxicity within 24 hr of exposure to lidocaine *in vitro*,^11^ and *in vivo*,^17^with concentrations as low as 0.5% inducing cytotoxicity and 1% resulting in near-complete cell death. Lidocaine-induced cell death appears to be related to apoptosis, and the toxic effects are more pronounced in osteoarthritic cartilage compared to healthy tissue.^33^ Although data on lidocaine toxicity in canine FLS remains limited, our findings show that even low concentrations can induce substantial cytotoxicity, highlighting potential intra-articular risks.

Similarly, mepivacaine exhibited clear dose-dependent toxicity, with 70% reduction of FLS viability at 4 mg/mL (0.4 %) and an estimated IC_50_ of approximately 1 mg/mL. Our results represent the first direct report of this drug species-specific effects in canine synoviocytes and are concordant with previous studies on cartilage cells of other species, where 1% mepivacaine significantly reduced cell viability of equine FLS and chondrocytes.^36^ Another study with equine FLS and chondrocytes revealed that after 24hr exposure to 4.4 mg/mL of mepivacaine negatively impacted cell viability with higher cytotoxicity towards chondrocytes than synoviocytes.^37^ Levobupivacaine, the s-isomer of racemic bupivacaine, is less toxic as a local anaesthetic compared to bupivacaine but equally effective,^38^ although direct studies evaluating levobupivacaine’s cytotoxicity in canine cartilage cells remain very limited. Our findings after 24-hour exposure to levobupivacaine in canine FLSs are consistent with previous reports in cartilage cells, showing that the highest concentration tested (0.6 mg/mL; 0.06%) reduced cell viability by 50%. An *in vitro* study with canine chondrocytes reported that an exposure to 0.062% levobupivacaine for 24hr significantly decreased chondrocyte viability and induced apoptosis through both the extrinsic and intrinsic pathways, as evidenced by increased caspase-3 activity.^11^ Previous studies with bupivacaine in canine chondrocytes and FLS obtained similar effects after 24hr exposure to 0.065% bupivacaine.^12^ Our findings provide the first direct evidence of levobupivacaine’s species-specific effects on canine FLS, addressing a significant gap in the literature.

Regarding corticosteroids, dexamethasone exhibits pronounced toxicity in canine FLSs, as even the lowest concentration tested (1 µg/mL) reduced cell viability by 50% and the lowest IC_50_ ≈ 3.4×10^-4^ mg/mL, providing the first report of dexamethasone’s toxic effects in canine synoviocytes. Previous studies with canine chondrocytes and tendon cells have shown that dexamethasone exhibits cytotoxic effects on canine joint cells in a dose- and time-dependent manner with concentrations ranging from 0.1 to 50 μg/ml significantly reduce the proliferation of these cells and increase the number of apoptotic nuclei, with higher concentrations (25-50 µg/mL) causing more pronounced effects.^12^ In contrast, low-dose sustained-release dexamethasone delivery using specialized implants has been shown to protect cartilage and improve functional recovery after osteochondral repair in canine models, while avoiding notable cytotoxic or inflammatory effects.^39^ Methylprednisolone and methylprednisolone acetate also exhibit dose and time-dependent cytotoxicity to articular cells across species.^40^ In equine fibrocartilage explants, 0.5mg/mL and 5mg/mL significantly reduced cell metabolic activity with increased cell death after 24hr.^33^ In canine cartilage and synovial explants, clinically relevant dose of 40 mg of methylprednisolone acetate exhibited significant chondrocyte and synoviocyte cytotoxicity within 24 hr in vitro^11^ and in vivo.^17^ Although direct evidence of these two corticosteroids cytotoxicity towards canine cartilage cells remains limited, our findings are aligned with previous reports, methylprednisolone exhibited a prevalent cytotoxicity above 50% inhibition of FLS metabolic activity at the highest concentrations of 5 mg/mL and 10 mg/mL. Methylprednisolone acetate exhibited a more prevalent cytotoxicity effect towards FLS with an IC_50_ ≈ 0.5 mg/mL.

In terms of inflammatory modulation, the tested IC_50_ concentrations for both the LAs and corticosteroids revealed no significant cytotoxicity effects on FLS and distinct modulatory profiles towards IL-6 expression and MMP-9 activity. Lidocaine, methylprednisolone and methylprednisolone acetate consistently supressed IL-6 expression to undetectable levels under TNF-α stimulation, while levobupivacaine exhibited lower reduction of IL-6 compared to control. Mepivacaine not only maintained high IL-6 levels under TNF-α stimulation (542.56 pg/mL) but was also the only compound to induce significant IL-6 expression (162.51 pg/mL) in the absence of TNF-α. This inherent pro-inflammatory potential of mepivacaine distinguishes it from lidocaine and is consistent with previous observations in equine synoviocytes where mepivacaine alone increased IL-6 and IL-1β mRNA expression.^41^ Existent literature on the modulation of IL-6 by LAs predominantly focuses on systemic or plasma IL-6 levels,^18-20,41^ with a gap of direct data concerning local joint or FLS expression. While lidocaine has been more extensively studied and generally shown to decrease IL-6 levels,^42^ specific impact on joint cells is largely unexplored. Notably, the direct effects of mepivacaine and levobupivacaine on IL-6 expression are also scarce. To address this gap, we report for the first time the modulation of IL-6 expression by these LAs within the joint context, specifically in OA-induced canine FLS, of which 3 mg/mL lidocaine was the most effective with complete inhibition of IL-6 expression. Corticosteroids are more well established regarding their anti-inflammatory effects, including IL-6 inhibition in articular tissues^25,26^ and the results obtained with methylprednisolone and methylprednisolone acetate confirm their anti-inflammatory effect. Dexamethasone, despite being a potent corticosteroid, resulted in a paradoxical increase in IL-6 expression compared to the TNF-α stimulated control. One plausible explanation for this observation is that the concentration of dexamethasone used (3.0 × 10^-4^ mg/mL) may have been insufficient to achieve effective IL-6 inhibition in this specific cell model, since the anti-inflammatory effects of dexamethasone are highly dose-dependent and can vary significantly across different cell types and inflammatory stimuli.^25-27^ In some *in vitro* models, particularly those involving FLS, relatively high concentrations of glucocorticoids are required to fully suppress the robust cytokine production induced by potent triggers like TNF-α.^43^

Regarding MMP-9 activity, upregulation of MMP-9 was observed in the TNF- α stimulated control, which is consistent with the established role of this gelatinase as a central mediator in the pathogenesis of OA.^9,10^ All tested agents demonstrated inhibitory effects with significant differences compared to control, in which dexamethasone emerged as the most potent inhibitor of MMP-9 by 70%. This differential regulation between IL-6 and MMP-9 observed with dexamethasone is likely due to the distinct signalling architectures and promoter sensitivities of these two mediators, suggesting that lower concentrations of dexamethasone can act as selective modulator in the canine synovial environment.^44^ Amongst the tested LAs, mepivacaine significantly inhibited 40% of MMP-9 activity. Previous studies have shown that mepivacaine can inhibit the activation of mitogen-activated protein kinases (MAPKs) and the subsequent translocation of transcription factors like AP-1, which are critical for MMP-9 gene expression.^33^ MMP-9 is a key enzyme in OA responsible for the degradation of extracellular matrix components within the joint that drive cartilage breakdown and synovial remodelling in OA.^9,10^ Despite its critical role, current evidence related to the effects of mepivacaine, levobupivacaine and methylprednisolone on MMP-9 remains scarce, especially within cartilage or synovial fibroblasts. Our results provide the first direct data in canine FLS under *in vitro* inflammatory conditions, filling a significant gap in the translational literature.

These findings underscore the complexity of intra-articular drug actions on synovial environment, which is highly sensitive and FLS play a crucial role in maintaining synovial fluid homeostasis and joint health.^11^ This study presents an exploratory *in vitro* toxicity screening of commonly used intra-articular drugs and inflammatory response of key mediators in OA, namely IL-6 and MMP-9. To our knowledge, this study provides the first evaluation of the *in vitro* toxicity of commonly used intra-articular LAs and corticosteroids in canine FLS including under inflammatory conditions. Given that canine OA is increasingly recognized as a translational model^6^ and the widespread clinical use of these agents, these findings offer novel and clinically relevant insights into how intra-articular therapies may influence the catabolic environment of the joint. This new data provides helpful insights that can directly inform clinical practice regarding the judicious selection and optimal dosing of intra-articular agents. Clinical implications of LAs and corticosteroids on synovial health are dependent on their pharmacokinetics and duration of action. LAs primarily block nerve impulses and are characterized by rapid onset and transient presence in the synovial fluid within minutes to hours.^45^ Lidocaine and mepivacaine concentrations in synovial fluid have been measured in horses, showing initial concentrations around 6–12 mg/mL for lidocaine and 5–8 mg/mL for mepivacaine within minutes after injection, with levels decreasing by about 33–60% after 23 minutes, of which these concentrations exceed those associated with cytotoxicity *in vitro*^46.^ Although direct canine joint pharmacokinetics for levobupivacaine are lacking, similar local anaesthetics show rapid decline in synovial fluid concentrations within the first hour post-injection, suggesting that effective concentrations diminish substantially over 24 hours.^46,47^ In contrast, corticosteroids exert anti-inflammatory effects through genomic pathways and are designed for prolonged synovial persistence. While dexamethasone clears relatively quickly, MPA maintains elevated concentrations in the synovial fluid for days to weeks.^48,49^This extended exposure to potentially cytotoxic concentrations poses a sustained risk for cumulative cellular damage, contrasting with the transient high-concentration risk of LAs. Therefore, optimizing dosage for both drug classes is essential, advocating for the lowest effective doses to mitigate joint cell damage.

Nevertheless, this study is subject to limitations inherent to its *in vitro* design which does not fully recapitulate the complex physiological environment of a living joint, combined with a 24hr static exposure period that cannot fully mimic the *in vivo* pharmacokinetics of each intra-articular agent. Additionally, only the individual effects of each agent were assessed, whereas in clinical practice these drugs are often administered in combination or sequentially. Additionally, the use of MTT assay to assess cell viability is an indirect approach based on the measurement of mitochondrial metabolic activity and reductions in signal may reflect metabolic suppression rather than direct cell death. Consequently, future work could incorporate complementary assays to better distinguish between metabolic inhibition, apoptosis and necrosis. Future research should include evaluation of other joint cell types, incorporation of dynamic *in vitro* models that better mimic *in vivo* clearance rates and explore the potential synergistic or antagonistic interactions between combined therapies. Despite these limitations, these findings provide a robust foundation for understanding the direct cellular effects of these intra-articular agents, informing future *in vivo* studies and clinical practice.

## Conclusions

In summary, this study provides novel insights into *in vitro* effects of commonly used intra-articular agents in canine FLS. Results demonstrate that LAs including lidocaine, mepivacaine, levobupivacaine, as well as corticosteroids dexamethasone, methylprednisolone and methylprednisolone acetate exert dose-dependent cytotoxic effects on canine FLS even at clinically relevant concentrations. Notably, Dexamethasone and levobupivacaine exhibited the highest cytotoxicity within their respective tested ranges. The estimated IC_50_ values provide important reference points for intra-articular administration, offering a scientific basis for optimizing dosages to minimize cellular damage in clinical practice. Importantly, this study presents the first evidence of mepivacaine, levobupivacaine, and dexamethasone-induced toxicity in canine synoviocytes, highlighting species-specific responses that are essential for translational research. Our findings also elucidate the complex and differential modulation of key inflammatory mediators IL-6 and MMP-9. Lidocaine, methylprednisolone and methylprednisolone acetate effectively suppressed TNF-α induced IL-6 expression while mepivacaine presented pro-inflammatory potential. Furthermore, dexamethasone demonstrated significant inhibitory potential of MMP-9 activity in contrast with IL-6 expression, suggesting a mediator-specific and concentration-dependant nature of this corticosteroid within the synovial environment. These findings underscore the critical importance of considering the direct cellular impact of intra-articular therapies. By providing detailed toxicity profiles and immunomodulatory data, this study offers valuable information that can directly inform clinical decision-making and the development of safer therapeutic strategies. Given the established translational value of the canine model for human osteoarthritis, these results also contribute to a broader understanding of joint health and disease across species, paving the way for more effective and targeted intra-articular treatments.
